# MiR‐29a‐3p suppresses cell proliferation in laryngocarcinoma by targeting prominin 1

**DOI:** 10.1002/2211-5463.12199

**Published:** 2017-03-18

**Authors:** Jili Su, Eryong Lu, Lijuan Lu, Chao Zhang

**Affiliations:** ^1^ Department of Otorhinolaryngology, Head and Neck SurgeryThe First Affiliated Hospital, and College of Clinical Medicine of Henan University of Science and TechnologyLuoyangChina

**Keywords:** laryngocarcinoma, miR‐29a‐3p, PROM1

## Abstract

MicroRNAs (miRNAs) are known to play a regulatory role in various cancers including laryngocarcinoma. MiR‐29a‐3p is a potential tumor‐suppressive miRNA, but its function in laryngocarcinoma is unknown. The purpose of this study was to investigate the roles of miR‐29a‐3p in laryngocarcinoma. Prominin1 (PROM1) was predicted as a target gene of miR‐29a‐3p and this was verified using a luciferase reporter assay. Transfection of miR‐29a‐3p into two laryngocarcinoma cell lines indicated that miR‐29a‐3p could decrease cell proliferation and enhance the chemotherapy response by targeting PROM1. PROM1 expression was up‐regulated in the laryngocarcinoma cells when miR‐29a‐3p was down‐regulated. We found miR‐29a‐3p expression levels were lower in laryngocarcinoma tissues than in control tissues. We also found that miR‐29a‐3p expression was negatively correlated with PROM1 expression in laryngocarcinoma tissues. The study demonstrates that miR‐29a‐3p suppresses cell proliferation in laryngocarcinoma by targeting PROM1.

**Abbreviations**
miRNAs: MicroRNAs
MTT: 3‐(4,5‐dimethylthiazol‐2‐yl)‐2,5‐diphenyl‐tetrazolium bromide
PROM1: prominin1
PVDF: polyvinylidene difluoride

Laryngocarcinoma is a common type of cancer and has a high incidence and mortality. The mortality of laryngocarcinoma is decreasing in countries with advanced healthcare systems, but the incidence is still high in developing countries 1, 2. Therefore, it is very important to elucidate the molecular pathogenesis mechanism of laryngocarcinoma progression in order to find new treatment strategies.

MicroRNAs (miRNAs) belong to a class of noncoding mRNA, around 22 nucleotides in length. There is accumulating evidence that miRNAs play an important role in gene expression regulation by specifically binding and cleaving mRNAs or inhibiting their translation 2, 3. Deepening the knowledge of laryngocarcinoma genomes will help to find new targets of therapy and understand the molecular pathology of the disease. In human laryngocarcinoma, a number of miRNAs have been reported to be aberrantly expressed during its progression, including miR‐210, miR‐205, miR‐301a‐3p, miR‐34c‐5p, miRNA‐221, miR‐21, miR‐519a etc. 4, 5, 6, 7, 8, 9, 10. MiR‐29a‐3p is reported as a tumor‐suppressing miRNA in breast and gastric cancers 11, 12, but its role in laryngocarcinoma is not clear.

In the present study, we hypothesize that miR‐29a‐3p acts as a tumor suppressor in laryngocarcinoma. We examine miR‐29a‐3p expression in laryngocarcinoma tissues and cell lines and test its effects on cell growth, colony formation, and drug response *in vitro*. We also explore the mechanism of regulation on miR‐29a‐3p in laryngocarcinoma cells.

## Materials and methods

### Laryngocarcinoma tissues

Informed consent was obtained from each patient, and the research protocols were approved by the Ethics Committee of Henan University of Science and Technology. Laryngocarcinoma tissues and their normal controls were obtained from The First Affiliated Hospital (Luoyang, China). Both tumor and normal tissues were histologically confirmed by H&E (hematoxylin and eosin) staining.

### Cell culture

Laryngocarcinoma cell lines including TU212, M4E, M2E, and Hep‐2 were maintained in Eagle's Minimum Essential Medium (Invitrogen, Carlsbad, CA, USA), supplemented with 10% FBS (HyClone, Logan, UT, USA) and 1% penicillin/streptomycin (Invitrogen). Normal nasopharyngeal epithelial cell line NP69 was primarily from ATCC and was maintained in the RPMI1640 medium containing 10% FBS and 1% penicillin/streptomycin (Invitrogen) at 37 °C, 5% CO_2_.

### Luciferase assay

For measuring the effect of miR‐29a‐3p on the 3′‐UTR of Prominin1 (PROM1), we generated a luciferase expression construct containing part of PROM1 3′‐UTR. We amplified the wild‐type fragment PROM1 mRNA that contained potential miR‐29a‐3p‐binding sites at position 534–540, using the following primers: 5′‐CTGAGTTTCTATTTAGACACTACAACA‐3′ (forward) and 5′‐ACAATTTGACATGTGGCATTAACG‐3′ (reverse). The PCR fragment was inserted into the pGL4 Basic Vector (Promega, Madison, WI, USA) using the *Kpn*I/*Xho*I endonuclease restriction sites. Mutation of the PROM1 3′‐UTR (Mut) was performed using a mutation kit (Stratagene, La Jolla, CA, USA). For luciferase activity assays, cells were cotransfected with 100 ng of wild‐type or Mut RROM1 3′‐UTR and 100 nm miR‐29a‐3p or control mimics using Lipofectamine 2000. Luciferase activity was assayed using luciferase assay kit from Promega referring to the manufacturer's protocol after 48 h transfection, luciferase activity was measured and normalized to Renilla luciferase activity.

### Real‐time RT‐PCR

Total RNA from the cells with miRNA mimics, inhibitors or PROM1 transfection was extracted using Trizol reagent (Invitrogen) following the manufacturer's instructions. After that, 2 μg of RNA was taken and treated with DNase to remove contaminating DNA prior to the reverse transcription to cDNA using SYBR® PCR Kit (Takara, Japan). To measure expression, real‐time RT‐PCR was performed using a sequence detector (ABI‐Prism, Applied Biosystems, Foster City, CA, USA). The relative expression levels were calculated using Ct method and all data were normalized to the internal controls.

### MTT assay

The proliferation of laryngocarcinoma cells was examined by 3‐(4,5‐dimethylthiazol‐2‐yl)‐2,5‐diphenyl‐tetrazolium bromide (MTT) assay. Simply, 2 × 10^3^ cells were seeded to a 96‐well plate. At different time points, 10 μL MTT solution (5 mg·mL^−1^, Sigma‐Aldrich, St. Louis, MO, USA) was added to each well and the cells were cultured for 4 h. After the incubation, the supernatant was discarded and 150 μL dimethyl sulfoxide was added to each well until crystal dissolved completely. The absorbance was measured using an ELISA reader.

### Apoptosis assay

Apoptosis analysis was performed using an Annexin V‐FITC Apoptosis Detection Kit II (BD Bioscience; San Jose, CA, USA) according to the manufacturer's instructions. Cells were analyzed by flow cytometry using a FACSCalibur flow cytometer (BD Bioscience). The data were analyzed using winmdi software (The Scripps Research Institute, La Jolla, CA, USA).

### Western blot analysis

Protein from the cells was collected, qualified and then separated by SDS/PAGE, and transferred to polyvinylidene difluoride (PVDF) membranes (Millipore, Bedford, MA, USA). The membranes were blocked in 5% nonfat dry milk, the membranes were incubated with primary antibodies overnight at 4 °C, and then incubated with secondary antibodies conjugated with horseradish peroxidase in TBS for 2 h at room temperature. Finally, protein bands were visualized using ECL system on X‐ray films.

### Statistical analysis

All analyses were performed using the spss 16.0 statistical software package (spss, Chicago, IL, USA) or Excel. Every experiment was completed independently at least three times. A *P* value < 0.05 was considered significant.

## Results

### MiR‐29a‐3p down‐regulates PROM1 in laryngocarcinoma cells

**Figure 1:**
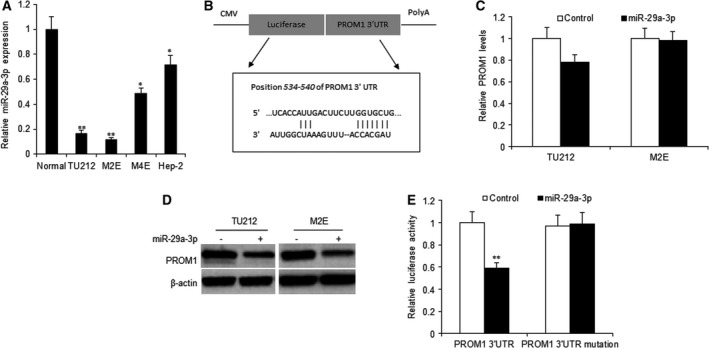
MiR‐29a‐3p down‐regulates PROM1 expression in laryngocarcinoma cells. (A) Real‐time PCR analysis of miR‐29a‐3p expression in four laryngocarcinoma cell lines and normal cells. (B) The 3′‐UTR of the PROM1 gene contains binding sites for miR‐29a‐3p according to bioinformatic analysis. (C) miR‐29a‐3p restoration down‐regulated PROM1 in laryngocarcinoma cells. Cells were transfected with miR‐29a‐3p or the control for 48 h, then collected for real‐time PCR. (D) miR‐29a‐3p restoration down‐regulated PROM1 in laryngocarcinoma cells. Cells were transfected with miR‐29a‐3p or the control for 48 h, then collected for western blotting analysis. (E) miR‐29a‐3p suppressed the expression of a luciferase reporter gene harboring the 3′‐UTR of PROM1. TU212 cells were transiently cotransfected miR‐29a‐3p and the indicated luciferase constructs, and luciferase activity was analyzed 48 h later. Data assessed from three independent experiments and the *P* values were calculated by *t*‐test (*P < 0.05; **P < 0.01).

Four human laryngocarcinoma cell lines were selected to detect miR‐29a‐3p expression. We found that in two laryngocarcinoma cell lines, miR‐29a‐3p expressed significantly lower than the normal cell line; however, in another two cell lines, miR‐29a‐3p expressed not much lower than the normal cells (Fig. 1A). To look for the target genes of miR‐29a‐3p in laryngocarcinoma cells, prediction software online [targetscan 7.0 (http://www.targetscan.org) and miRBase (http://www.mirbase.org)] was used to search for the most promising potential target genes. The findings showed that PROM1 was a potential target gene of miR‐29a‐3p (Fig. 1B). To determine whether PROM1 expression in laryngocarcinoma cells is regulated by miR‐29a‐3p, TU212 and M2E cells were transiently transfected with miR‐29a‐3p mimics and real‐time RT‐PCR or western blotting were carried out. The data showed that endogenous PROM1 mRNA levels decreased in the TU212 cells but not M2E cells transfected with miR‐29a‐3p (Fig. 1C). PROM1 protein decreased in the above two cell lines with miR‐29a‐3p (Fig. 1D). Results from luciferase activity assay showed that the luciferase activity of PROM13′UTR (wild‐type) in TU212 cells was suppressed by miR‐29a‐3p, and was unchanged for the mutated PROM1 3′UTR (Fig. 1E).

### MiR‐29a‐3p decreases laryngocarcinoma cell proliferation

**Figure 2:**
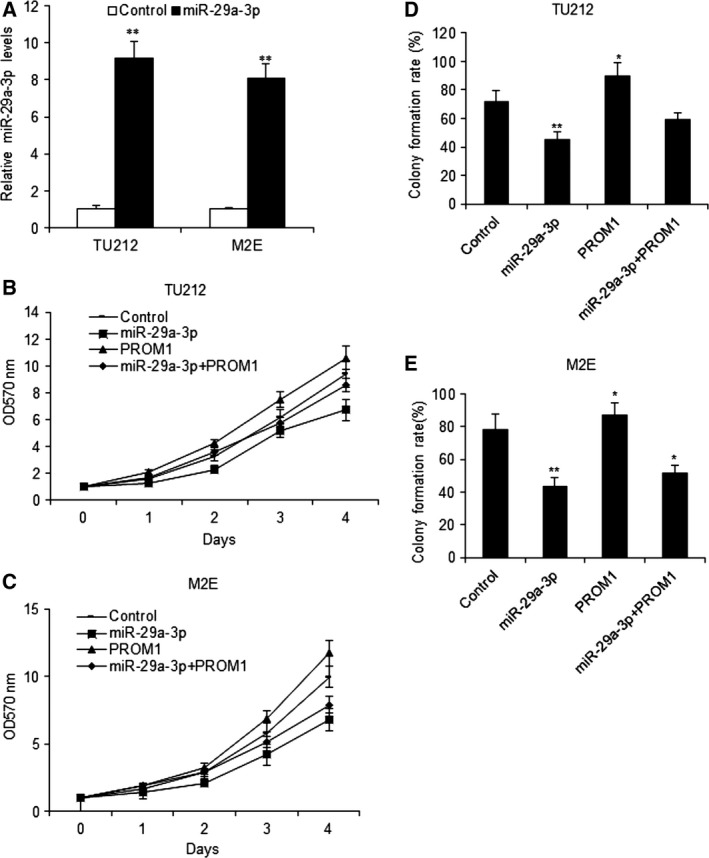
MiR‐29a‐3p decreases laryngocarcinoma cell growth *in vitro*. (A) miR‐29a‐3p expression in TU212 and M2E cells with miR‐29a‐3p mimics. (B) Effect of miR‐29a‐3p on cell proliferation was measured by MTT assay after miRNA infection in TU212 cells. (C) Effect of miR‐29a‐3p on cell proliferation was measured by MTT assay after miR‐29a‐3p and PROM1 transfection in M2E cells. (D) Effect of miR‐29a‐3p on cell proliferation was measured by colony formation assay after miR‐29a‐3p and PROM1 transfection in TU212 cells. (E) Effect of miR‐29a‐3p on cell proliferation was measured by colony formation assay after miR‐29a‐3p and PROM1 transfection in M2E cells. Data assessed from three independent experiments and the *P* values were calculated by *t*‐test (*P < 0.05; **P < 0.01).

To explore the cellular function of miR‐29a‐3p in laryngocarcinoma, TU212 and M2E cells were transfected with miR‐29a‐3p or the control and miR‐29a‐3p increased in the two cell lines (Fig. 2A). MTT assay was used to examine the cell proliferation, the data showed that miR‐29a‐3p could inhibit cell growth in TU212 and M2E cells (Fig. 2B,C). We used colony formation assay to evaluate cell proliferation, and the results showed that miR‐29a‐3p could significantly decrease colony formation rates in the above two cell lines (Fig. 2D,E).

### MiR‐29a‐3p enhanced the drug responses of laryngocarcinoma by PROM1

**Figure 3:**
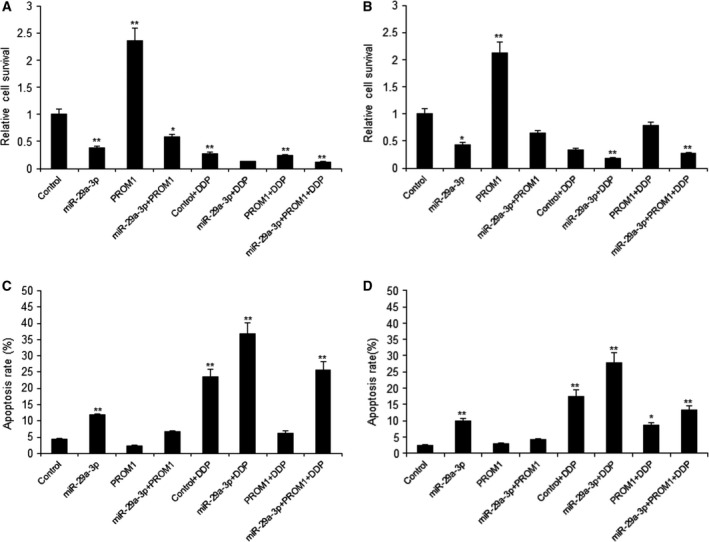
MiR‐29a‐3p enhanced the drug responses of laryngocarcinoma by PROM1. (A–B) Effect of miR‐29a‐3p on cell proliferation was measured by MTT assay after miRNA infection in TU212 and M2E cells and DDP treatment (10 μm). (C–D) miR‐29a‐3p promoted laryngocarcinoma cell apoptosis by PROM1. TU212 and M2E cells were transfected with miR‐29a‐3p or PROM1 lentivirus and then treated with DDP (10 μm). Cell apoptosis was analyzed by flow cytometry. Data assessed from three independent experiments and the *P* values were calculated by *t*‐test (*P < 0.05; **P < 0.01).

Data from Fig. 1 showed that PROM1 was a direct target gene of miR‐29a‐3p, we proposed that miR‐29a‐3p is related to the chemotherapy response via PROM1. In order to elucidate the question, TU212 cells were transfected with miR‐29a‐3p or PROM1 vector, cell survival was assayed by MTT in cells with DDP treatment. The results indicated that miR‐29a‐3p increased the therapy effect in laryngocarcinoma cells (Fig. 3A,B). It was also demonstrated that miR‐29a‐3p promoted cell apoptosis in the cells with drug treatment (Fig. 3C,D).

### MiR‐29a‐3p expression in clinical laryngocarcinoma samples

**Figure 4:**
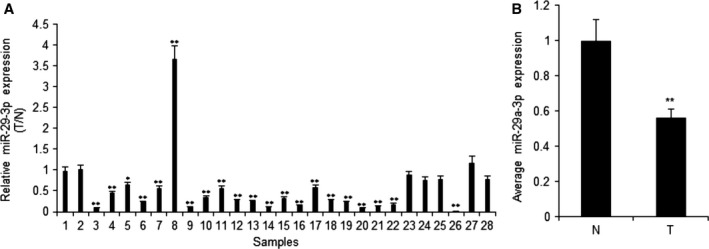
MiR‐29a‐3p expression in human laryngocarcinoma. (A) Relative miR‐29a‐3p expression levels in laryngocarcinoma tissues and their corresponding adjacent normal tissues. MiR‐29a‐3p expression was assessed by real‐time RT‐PCR. T, laryngocarcinoma tissues; N, adjacent normal tissues. (B) Average expression level of miR‐29a‐3p in cancer tissues or the normal control tissues. T, laryngocarcinoma tissues; N, adjacent normal tissues. Data assessed from three independent experiments and the *P* values were calculated by *t*‐test (*P < 0.05; **P < 0.01).

Firstly, the expression of miR‐29a‐3p in human laryngocarcinoma specimens was examined by real‐time RT‐PCR. The levels of miR‐29a‐3p were lower in laryngocarcinoma tissues than the normal samples (Fig. 4A). The levels of miR‐29a‐3p in laryngocarcinoma tissues were analyzed and the result showed that the levels of miR‐29a‐3p in the tumor tissues were lower than in the normal tissues (Fig. 4B). These results suggested that miR‐29a‐3p may act as an inhibitor in the progression of human laryngocarcinoma.

### MiR‐29a‐3p is negatively related to PROM1 expression in laryngocarcinoma

**Figure 5:**
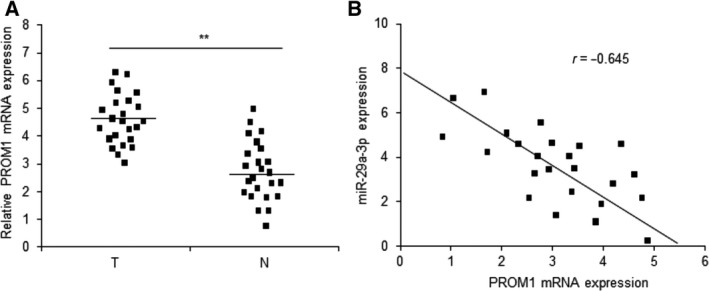
Relationship of miR‐29a‐3p and PROM1 in laryngocarcinoma. (A) PROM1 expression in laryngocarcinoma tissues was higher compared to their adjacent normal tissues by qRT‐PCR assay. T: laryngocarcinoma tissues; N: adjacent normal tissues. (B) miR‐29a‐3p was negatively associated with PROM1 mRNA in laryngocarcinoma tissues. Data assessed from three independent experiments and the *P* values were calculated by *t*‐test (**P < 0.01).

The above data suggested that miR‐29a‐3p decreased laryngocarcinoma cell proliferation and enhanced drug responses by targeting PROM1. In order to investigate PROM1 expression in laryngocarcinoma tissues, the data from qRT‐PCR showed that PROM1 mRNA was more up‐regulated in laryngocarcinoma tissues than their normal ones (Fig. 5A). The clinic data showed that miR‐29a‐3p was negatively associated with PROM1 in laryngocarcinoma tissues (Fig. 5B).

## Discussion

Many miRNAs are discovered in laryngeal cancers and they act as important regulators in gene expression at post‐transcriptional levels; however, there are still unknown miRNAs. Previous reports indicated that miR‐29a‐3p was a tumor‐suppressive miRNA in some types of cancer 11, 12. We found that the expression of miR‐29a‐3p was significantly down‐regulated in laryngocarcinoma tissues or cell lines compared with the normal controls. Introduction of miR‐29a‐3p expression in laryngocarcinoma cells decreased cell proliferation and increased drug sensitivity. PROM1, an oncogene, was verified as a direct target gene of miR‐29a‐3p in laryngocarcinoma cells. Our data for the first time demonstrated that miR‐29a‐3p inhibited laryngocarcinoma cell survival by targeting PROM1, which suggests that miR‐29a‐3p functions as a tumor‐suppressive miRNA.

Most previous studies indicate that miR‐29a‐3p functions as a tumor‐suppressing molecule in breast cancer 11 and gastric cancer 12. MiR‐29‐3p includes miR‐29a‐3p, miR‐29b‐3p, and miR‐29c‐3p. Our study focused on miR‐29a‐3p due to its low expression in laryngocarcinoma. There were no significant differences of miR‐29b‐3p and miR‐29c‐3p levels in laryngocarcinoma cells and normal cells. Our study identified that miR‐29a‐3p expression in laryngocarcinoma tissues was down‐regulated compared to their matched normal adjacent tissues, which was also confirmed in laryngocarcinoma cells. A lack in the expression of miR‐29a‐3p was also significantly correlated with the metastasis of laryngocarcinoma in clinical specimens. In the face of cellular functions, miR‐29a‐3p decreased cell viability, colony formation, proliferation, and promoted apoptosis in laryngocarcinoma cells. So, all the data suggest that miR‐29a‐3p acts as an inhibiting miRNA in laryngocarcinoma.

Prominin1 plays an important role in the initiation and progression of tumors 13, 14, 15. PROM1 was a marker of stem cell or a potential diagnostic molecule in cancers including nonsmall cell lung cancer 16, 17, glioma 18, medulloblastoma 19, and glioblastoma 20. In this study, we confirmed that PROM1 was a downstream target of miR‐29a‐3p, and it was also a functional mediator of miR‐29a‐3p in laryngocarcinoma cells. There was a complementary sequence of miR‐29a‐3p in the 3′‐UTR of PROM1, which was verified the luciferase activity was inhibited, but there was no influence on the mutant PROM1 3′‐UTR when laryngocarcinoma cells were transfected with miR‐29a‐3p or the wild‐type of PROM1 3′‐UTR. It suggested miR‐29a‐3p might be combined with PROM1 3′‐UTR and then suppressed PROM1 transcription. Further study demonstrated that miR‐29a‐3p regulated PROM1 expression at the post transcriptional levels.

In summary, we identified that miR‐29a‐3p was a suppressing miRNA in laryngocarcinoma. MiR‐29a‐3p partially influences human laryngocarcinoma through the regulation of PROM1. These results suggest that miR‐29a‐3p is a potential target for treating laryngocarcinoma and the critical roles of miR‐29a‐3p in laryngocarcinoma tumorigenesis may help patient prognosis and diagnosis. Our findings provide basic information to better understand the pathogenesis of laryngocarcinoma and its possible therapeutic strategies.

## Author contributions

JS and CZ conceived and designed the project and wrote the paper; JS, EL, and LL acquired the data; and JS and EL analyzed and interpreted the data.
